# Study of major genetic factors involved in pituitary tumorigenesis and their impact on clinical and biological characteristics of sporadic somatotropinomas and non-functioning pituitary adenomas

**DOI:** 10.1590/1414-431X20187427

**Published:** 2018-06-25

**Authors:** R.K. Foltran, P.V.G.H. Amorim, F.H. Duarte, I.P.P. Grande, A.C.T.B. Freire, F.P. Frassetto, J.B. Dettoni, V.A. Alves, I. Castro, E.B. Trarbach, M.D. Bronstein, R.S. Jallad

**Affiliations:** 1 Laboratorio de Endocrinologia Celular e Molecular, LIM25, Disciplina de Endocrinologia, Hospital das Clínicas, Faculdade de Medicina, Universidade de São Paulo, São Paulo, SP, Brasil; 2 Unidade de Neuroendocrinologia, Disciplina de Endocrinologia, Hospital das Clínicas, Faculdade de Medicina, Universidade de São Paulo, São Paulo, SP, Brasil; 3 Divisao de Anatomia Patológica, Hospital das Clínicas, Faculdade de Medicina, Universidade de São Paulo, São Paulo, SP, Brasil; 4 Divisao de Medicina Molecular, Departamento de Medicina, Hospital das Clínicas, Faculdade de Medicina, Universidade de São Paulo, São Paulo, SP, Brasil; 5 Serviço de Endocrinologia, A.C. Camargo Center, São Paulo, SP, Brasil

**Keywords:** Somatotropinomas, Non-functioning pituitary adenomas, PTTG, AIP, GNAS1

## Abstract

Genetic and functional aberrations of guanine nucleotide-binding protein, alpha stimulating (*GNAS*), aryl hydrocarbon receptor interacting protein (*AIP*), and pituitary tumor transforming gene (*PTTG*) are among the most prominent events in pituitary tumorigenesis. A cohort of Brazilian patients with somatotropinomas (n=41) and non-functioning pituitary adenomas (NFPA, n=21) from a single tertiary-referral center were evaluated for *GNAS* and *AIP* mutations and gene expression of *AIP* and *PTTG*. Results were compared to the clinical and biological (Ki67 and p53 expression) characteristics of tumors and their response to therapy, if applicable. Genetic analysis revealed that 27% of somatotropinomas and 4.8% of NFPA harbored *GNAS* mutations (P=0.05). However, no differences were observed in clinical characteristics, tumor extension, response to somatostatin analog therapy, hormonal/surgical remission rates, Ki67 index, and p53 expression between mutated and non-mutated somatotropinomas patients. *PTTG* overexpression (RQ mean=10.6, min=4.39, max=11.9) and *AIP* underexpression (RQ mean=0.56, min=0.46-max=0.92) were found in virtually all cases without a statistically significant relationship with clinical and biological tumor features. No patients exhibited somatic or germline pathogenic *AIP* mutations. In conclusion, mutations in *GNAS* and abnormal *PTTG* and *AIP* expression had no impact on tumor features and treatment outcomes in this cohort. Our data support some previous studies and point to the need for further investigations, probably involving epigenetic and transcriptome analysis, to improve our understanding of pituitary tumor behavior.

## Introduction

Pituitary adenomas are mostly sporadic and result from genetic mutations in a single cell involving overexpression of oncogenes, inactivation of tumor suppressor genes, and alterations of transcription factors regulating cell growth and differentiation (1,2). An abnormal response to hypothalamic stimulation can provide a permissive environment for molecular events to occur within the pituitary gland. However, the precise mechanisms of tumorigenesis remain unclear (1,2).

Proto-oncogenes *GNAS* (guanine nucleotide-binding protein, alpha stimulating), *PTTG* (pituitary tumor-transforming gene 1), and tumor suppressor gene *AIP* (aryl-hydrocarbon receptor-interacting protein) are the most prominent genetic factors involved in pituitary tumorigenesis and have been implicated in the development of somatotropinomas and non-functioning pituitary adenomas (NFPA) (2). In addition, a recent meta-analysis study points to a putative role for *GNAS1* mutation as a prognostic factor of treatment response to somatostatin receptor ligands (3). Nevertheless, the importance of *GNAS1*, *PTTG*, and *AIP* as molecular markers in the prognosis of pituitary adenomas is still controversial (4–7).

In this study, we assessed the presence of *GNAS* and *AIP* mutations as well as altered gene expressions of *AIP* and *PTTG* in sporadic somatotropinomas and NFPA patients admitted to a single endocrine tertiary-referral center. The importance of these genetic factors, Ki-67 cell proliferation marker, and p53 on the prognosis of pituitary adenomas was also evaluated.

## Material and Methods

Patient information regarding age, hormone levels, family background, and tumor characteristics (size and extension) at diagnosis was retrospectively obtained from medical records. Histopathologic analyses and follow-up data after surgery were recorded for each patient. After surgery, clinical, hormonal, and radiological data were evaluated. Tumor size was measured as the maximum diameter on MRI and tumors were classified accordingly as microadenomas (<10 mm) or macroadenomas (≥10mm). Treatment and follow-up were based on physician discretion in accordance with current guidelines (8). For all patients, transsphenoidal surgery was the primary therapy for acromegaly. No patient underwent radiotherapy. After non-effective pituitary surgery, somatostatin receptor ligand (octreotide LAR) and/or dopamine agonist (cabergoline) were used in acromegalic patients to achieve hormonal and/or tumor control. The last follow-up data, including patients treated with different therapies, were analyzed to define clinical status regarding remission and disease activity. For acromegaly, controlled disease criteria were normal age-adjusted insulin-like growth factor-1 (IGF-1) concentrations and a random growth hormone (GH) <1ng/mL. For NFPAs, tumor control was based on either the complete resection of tumor, size stability or reduction of the lesion on MRI; in cases with incomplete primary resection, tumor stability was assessed in terms of tumor remnant behavior.

This study was approved by the Hospital das Clínicas, Faculdade de Medicina, Universidade de São Paulo ethics committee and all patients signed an informed consent form.

### Extraction of nucleic acid

DNA and RNA were extracted from fresh tumor cryosections using All Prep DNA/RNA Mini Kit (Qiagen, USA) according to manufacturer's protocol. DNA and RNA were used for genetic mutations and gene expression analysis, respectively. DNA extraction from blood was performed using DNeasy blood kit (Qiagen) according to manufacturer's protocol.

### Sanger sequencing and *in silico* analysis

All coding regions of AIP and hotspots of exons 8 and 9 of *GNAS* were amplified by polymerase chain reaction (PCR) as previously described, using tissue and tissue/germline DNA, respectively (9,10). Sequences obtained were compared with sequences in the Ensemble database (AIP, ENST00000279146 and GNAS, ENST00000371100) using Sequencher 3.0 software (Gene Codes, USA).

All variants identified were confirmed in two independent PCR products and sequencing of both strands. *In silico* predictions were performed only for rare variants using NetGene2 (http://www.cbs.dtu.dk/services/NetGene2/) and Human Splicing Finder (HSF, www.umd.be/HSF3).

### RNA expression

cDNA was synthesized using 1 µg of RNA by QuantiTect Reverse Transcription (Qiagen) following manufacturer's instructions. RNA quantification was performed in Step One Plus™ Real-Time PCR system (Applied Biosystems, USA) using PTTG (Hs00864094_g1) and AIP (Hs00610222_m1) TaqMan¯ assays (Applied Biosystems) according to the manufacturer's instructions. Relative quantification was calculated by 2^-ΔΔCT^ method using *HPRT1* as an endogenous gene (Hs02800695_m1, Applied Biosystems) and a commercial pool of RNA from human pituitary gland (Clontech, USA) as calibrator (11).

### Immunohistochemistry

Standard immunohistochemical reactions were carried out on formalin-fixed paraffin-embedded tissue to evaluate the presence of anterior pituitary hormones (GH, PRL, LH, FSH, TSH, and ACTH), Ki-67 (anti-human antigen Ki67, clone MIB-1, DAKO, cod. M7240, Denmark), and p53 (anti-human p53 protein, clone 318-6-11, DAKO, cod. M3629). For pituitary hormones, immunostaining was routinely performed in the Departmento de Patologia, Divisão do Laboratório Central, Hospital das Clínicas, Universidade de São Paulo (Brazil). The Ki-67 proliferative index was determined as the percentage of labeled nuclei cells. Ki67 and p53 nuclear staining was assessed in approximately 100 cells in 5 randomly chosen visual fields at ×400 magnification. The Ki-67 index was calculated from the mean of stained cells and considered high if >3 (12 [Bibr B13]). p53 expression was considered positive in patients with more than 10% of stained cells. These analyses were blinded and performed by two independent pathologists.

### Statistical analysis

Continuous variables were tested for normality with the Kolmogorov-Smirnov and Shapiro Wilk tests, and are reported as means±SD and median (25^th^ and 75^th^ percentiles) according to parametric or non-parametric distribution, respectively. Parametric data were compared using ANOVA. Non-parametric data were compared using the Mann-Whitney *U* test for two independent samples or Kruskal-Wallis test with Dwass-Steel-Chritchlow-Fligner *post hoc* test for three or more samples. Categorical data were analyzed using Pearson’s chi-squared test and Fisher’s exact test where applicable, and are reported as absolute values or percentages. Correlations were calculated using the Spearman rank test. Statistical significance was considered to be P≤0.05. Analyses were performed using SPSS 19.0 (IBM Analytics, USA) and Stata/SE 14.2 (StataCorp LLC, USA).

## Results

### Patient characteristics

Sixty-two patients with apparently sporadic pituitary adenomas (41 somatotropinomas and 21 NFPA) were evaluated. All patients' clinical symptoms were consistent with the diagnostic criteria that was confirmed by clinical, imaging, and pathology data related to each pituitary tumor subtype. Table 1 shows a summary of clinical, molecular, and immunohistochemical data of the somatotropinomas and NFPA patients evaluated.

**Table 1.** Summary of clinical, molecular, and immunohistochemical data of patients with somatotropinomas and non-functioning pituitary adenomas (NFPA) evaluated in this study.

	Somatotropinomas	NFPA	Total
Gender (female:male)	27:14	14:7	41:21
Age at diagnosis	40.3±15.3	48.4±11.0	42.9±14.4
Basal GH (ng/mL)	18.9 (6.2–75.4)	NA	NA
Basal IGF-1 (ng/mL)	964.5±280.5	NA	NA
ULNR-IGF-1 %	374.0 (297.0–435.0)	NA	NA
Tumor size			
Largest diameter	2.27±1.21	2.96±1.05	2.49±1.21
Micro x macro	4:37	0:21	4:58
Invasiveness (yes:no)	25:16	12:9	37:25
Tumor remission (yes:no)	11:20	11:3	30:13
Hormonal remission (yes:no)	26:15	NA	NA
*GNAS* mutations			
p.R201C	9	1	10
p.Q227L	1	0	1
p.Q227R	1	0	1
*AIP* mutations	0	0	0
*AIP* RQ	0.62 (0.43–0.85)	0.53 (0.51–1.28)	0.56 (0.46–0.92)
*PTTG* RQ	7.77 (4.39–11.9)	12.4 (10.1–16.2)	10.6 (4.39–11.9)
Ki67 (%)*	1.32 (1–4.5)	1.24 (1–3.8)	1.32 (1–4.5)
p53 (%)*	1.0 (1–1.4)	1.1 (1–1.8)	1.1 (1–1.8)

Data are reported as means±SD and median (25^th^ and 75^th^ percentiles). *Mean, minimum, and maximum values are reported for Ki67 index and p53. GH: growth hormone; IGF-1: insulin-like growth factor-I; ULNR: upper limit of the normal age- and sex-matched range; *GNAS*: guanine nucleotide-binding protein, alpha stimulating; *AIP*: aryl hydrocarbon receptor interacting protein; *PTTG*: pituitary tumor transforming gene; RQ: relative quantification; NA: not applicable.

### *GNAS* mutations

*GNAS* (p.Q227L, n=1; p.Q227R, n=1 and p.R201C, n=9) somatic missense mutations were found in heterozygosis in 11/41 patients with somatotropinomas (27%) and in 1/21 (p.R201C) with NFPA (4.8%) (P=0.05). Comparing the clinical and laboratory characteristics of patients with somatotropinomas harboring (+) or not (−) *GNAS* mutations, no significant difference was identified in gender, age of diagnosis, tumor size and extension, and hormonal and tumor remission (Table 2). Expression of *PTTG* and *AIP* mRNA and Ki-67 and p53 proteins also did not show significant differences between somatotropinomas *GNAS*+ and *GNAS*- (Table 2).

**Table 2.** Characteristics of mutated versus non-mutated *GNAS* patients with somatotropinomas.

	GNAS+	GNAS−	P value
Sex (female:male)	7:4	20:10	0.856
Age at diagnosis (years)	37.0±11.9	41.5±16.3	0.409
Basal GH (mg/dL)	37.4 (10.9–76.0)	15.1 (5.4–69.0)	0.233
Basal IGF-1 (mg/dL)	1004.9±257.5	949.7±291.3	0.583
ULNR IGF-1	331.0 (272.0–392.7)	388.0 (300.0–435.0)	0.377
Tumor size			
Largest diameter	2.36±1.05	2.23±1.29	0.771
Micro:macro	1:10	3:27	0.930
Invasiveness (yes:no)	8:3	17:13	0.478
Tumor expansion			
Intrasellar	2	7	0.970
Infrasellar+supra/parasellar	4	9	
Parasellar	1	2	
Suprasellar	1	6	
Para+suprasellar	3	6	
Remission (yes:no)			
After SST analogs	3:3	10:9	1.000
After surgery	4:6	7:14	0.358
*AIP* RQ	0.56 (0.47–0.95)	0.63 (0.36–1.00)	0.777
*PTTG* RQ	7.77 (5.39–12.8)	7.0 (3.58–11.7)	0.364
Ki67 index (%)*	1.7 (1.0–4.5)	1.33 (1.0–3.8)	0.297
p53 (%)*	1.1 (1.0–1.4)	1.00 (1.0–1.0)	0.083

Data are reported as means±SD and median (25^th^ and 75^th^ percentiles). *Mean, minimum, and maximum values are reported for Ki67 index and p53. GH: growth hormone; IGF-1: insulin-like growth factor-1; ULNR: upper limit of the normal age- and sex-matched range; *GNAS*: guanine nucleotide-binding protein, alpha stimulating; *AIP*: aryl hydrocarbon receptor interacting protein; *PTTG*: pituitary tumor transforming gene; RQ: relative quantification; SST: stomatostatin. ANOVA, Mann-Whitney *U* test, Kruskal-Wallis test, Pearson’s chi-squared test, and Fisher exact test were used where applicable.

### *AIP* variants

We found a previously described splicing variant c.468+15C>T (rs267607274, CS0910309) in the tumor's DNA from a patient with NFPA. This patient was diagnosed at 43 years old and had a null cell macroadenoma extending to the suprasellar region. *In silico* analysis using HSF) predicted a creation of a new intronic splicing enhancer, but both HSF and NetGene2 tools predicted no probable impact on splicing. Three other AIP polymorphic variants rs641081, rs2276020, and rs35665586 were found in germline and somatic DNA with a minor allelic frequency of 0.171, 0.025, and 0.005, respectively, similar to NCBI data bank. No evidence of AIP loss of heterozygosity was observed.

### *PTTG* and *AIP* mRNA expression

**Figure 1.**
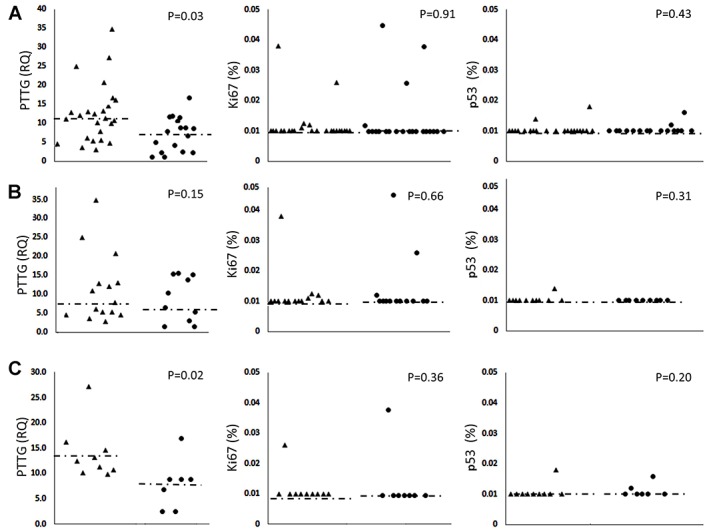
Expression of histological and molecular markers of proliferation/invasion in invasive and non-invasive pituitary adenomas. Graphs represent distributional plot of qPCR results for pituitary tumor transforming gene (*PTTG*) and immunohistochemical analysis (Ki67 and p53), with the scoring for the respective marker depicted in the y-axis. *A*, Somatotropinomas and non-functioning pituitary adenomas (NFPA). *B*, Somatotropinomas. *C*, NFPA. Dashed lines: medians; triangles: invasive cases; circles: non-invasive cases. RQ: relative quantification. Mann-Whitney *U* test was used for statistical analyses.

Gene expression analysis was performed in 43 samples (somatotropinoma, n=29, NFPA, n=14) with satisfactory RNA quality. *PTTG* expression level was significantly higher in NFPA compared to somatotropinomas (P=0.04, Table 1) and was associated with tumor invasiveness (P=0.03), especially in NFPA (P=0.02; Figure 1). There was no difference in the expression of *PTTG* and *AIP* in both somatotropinomas and NFPA, and hormonal dosage, age of diagnosis, tumor size, tumor and/or hormonal control, Ki-67 index, and p53 expression (data not shown).

### Ki-67 and p53 immunohistochemistry

Tumor samples for immunohistochemical staining were available from 45 patients (somatotropinoma, n=27, NFPA, n=18). This analysis showed a Ki-67 labeling index mean of 1.32% (1.0–4.5) and of 1.1% (1.0–1.8) for p53 expression. No difference was observed in the Ki-67 and p53 expression levels between somatotropinomas and NFPAs (P=0.27 and P=0.21, respectively, Table 1). In both somatotropinomas and NFPAs, Ki-67 and p53 expressions were not related with patient's age and tumor characteristics such as tumor size, infra-, intra-, para-, and suprasellar extension (data not shown). Neither Ki-67 nor p53 expression were correlated with hormonal levels and radiological recurrence in somatotropinomas and NFPAs, respectively, or tumor invasiveness (Figure 1B).

## Discussion

The present study evaluated clinical, laboratory, and *GNAS*, *AIP*, and *PTTG* molecular data of 62 apparently sporadic pituitary adenomas followed in a single endocrine tertiary-referral center. In line with previous results, *GNAS* proto-oncogene-activating mutations were the most frequent genetic mutation identified in 27 and 4.8% of somatotropinomas and NFPA, respectively (9,12). Comparing clinical and laboratory data of *GNAS*+ and *GNAS*- somatotropinomas patients, no significant difference was observed. These findings are similar to some data in the literature (7,14–17). However, other studies associated the presence of *GNAS* mutation with smaller (in diameter) and less invasive somatotropinomas (18–20).

The lack of phenotype-genotype correlation between *GNAS*-mutated and non-mutated pituitary adenomas could be explained by modulation of phosphodiesterase enzymes responsible for the hydrolysis of cyclic adenosine monophosphate (21). Persani et al. demonstrated that *GNAS*+ somatotropinomas had an increased activity of phosphodiesterase isoforms in relation to *GNAS*- somatotropinomas (21). Also, the *GNAS* locus displays a complex genomic imprinting being expressed only from the maternal allele in the pituitary gland (22). Some studies have demonstrated that *GNAS* loss of methylation at maternal promoter is associated to response to somatostatin analogs and contributes to the pathogenesis of acromegaly (23,24).

Regarding the *AIP* tumor suppressor gene, we found a very rare c.468+15C>T polymorphic *AIP* variant in a patient with NFPA. The c.468+15C>T was previously described in a young female patient with acromegaly due to a macroadenoma (24). According to *in silico* predictions, the c.468+15C>T variant has a lower probability to be deleterious. However, considering our results and previous published results, the calculated minor allele frequency (MAF) of this variant in pituitary adenomas was 0.004, much higher than MAF=0.000025 described by Exome Aggregation Consortium (ExAC; http://exac.broadinstitute.org/) (25,26). Therefore, we speculate that this noncoding nucleotide change could be related with pituitary tumorigenesis of somatotropinomas and NFPA. Additional analyses are essential to determine the real significance of this variant for *AIP* function.

Usually, *AIP* loss-of-function mutations are associated with a more aggressive disease and a low probability of surgical remission (27,28). These mutations are uncommon in sporadic pituitary adenomas, but their prevalence may increase among young patients: 23.3% (age at diagnosis ≤18 years) and 20.5% (age at diagnosis ≤30 years) (9,29,30). In our study, 34% of acromegalic patients were diagnosed ≤30 years old. Comparing young patients with patients older than 30 years at diagnosis, there was no difference in hormone assessment, tumor size, and response to treatment. Nevertheless, we have not identified *AIP* mutations in these patients, only very common polymorphic variants. mRNA expression was also evaluated and underexpression of *AIP* was found in virtually all patients, including in NFPA, but without any correlation with clinical and biological tumor features.

The *PTTG* expression observed in our cohort was high in almost all patients and was related significantly to invasiveness (P=0.03), particularly for NFPA (P=0.02). In fact, a recent meta-analysis study confirmed high expression levels of *PTTG* in different pituitary tumors and its correlation with invasiveness and tumor recurrence (31). However, no difference of *PTTG* levels between invasive and non-invasive somatotropinomas was found in our study (P=0.141). Although some studies showed a higher *PTTG* expression in acromegalic patients than in NFPA (31,32 [Bibr B33]), we noted a significantly higher expression of *PTTG* in NFPA (P=0.04), which was in agreement with the higher rate of invasiveness of this tumor subtype (5,34,35).

In the current study, the Ki-67 cell proliferation marker showed positivity of less than 3% and the immunopositivity of p53 was not statistically significant. These results are in agreement with the literature, in which the majority of pituitary adenomas are typically 3% (35). Immunoexpression of both markers, p53 and Ki-67, was not different between NFPA and somatotropinomas. In addition, the level of expression of Ki-67 and p53 did not show any significant relationship with sex, age (below and above 30 years), characteristics of pituitary adenomas, (type, size, extension, infra- or suprasellar), residual tumor after surgery, and/or hormonal recurrence. These results are in contrast to some studies in which the simultaneous expression of the p53 and Ki-67 markers is related to invasion and tumor aggressiveness, as well as to tumor progression and/or recurrence of pituitary adenomas (6,12,36,37). The relationship between the expression of the p53 protein or Ki-67 cell proliferation marker with the pituitary adenoma has been studied (37,38). However, studies have not clearly shown the importance of Ki-67 and p53 immunohistochemical in the evaluation of predictive and prognostic factors of pituitary adenomas (38,39).

In conclusion, our results are in agreement with previous results that point to *PTTG* expression as a useful molecular marker for pituitary tumor invasiveness, particularly for NFPA. However, we did not find any evidence for the use of *GNAS* mutation or immunohistochemical Ki-67 and/or p53 staining as a potential marker to distinguish pituitary tumor behavior.

## References

[1] Aflorei ED, Korbonits M (2014). Epidemiology and etiopathogenesis of pituitary adenomas. J Neurooncol.

[2] Jiang X, Zhang X (2013). The molecular pathogenesis of pituitary adenomas: an update. Endocrinol Metab (Seoul).

[3] Efstathiadou ZA, Bargiota A, Chrisoulidou A, Kanakis G, Papanastasiou L, Theodoropoulou A (2015). Impact of gsp mutations in somatotroph pituitary adenomas on growth hormone response to somatostatin analogs: a meta-analysis. Pituitary.

[4] McCabe CJ, Khaira JS, Boelaert K, Heaney AP, Tannahill LA, Hussain S (2003). Expression of pituitary tumour transforming gene (PTTG) and fibroblast growth factor-2 (FGF-2) in human pituitary adenomas: relationships to clinical tumour behaviour. Clin Endocrinol (Oxf).

[5] Noh TW, Jeong HJ, Lee MK, Kim TS, Kim SH, Lee EJ (2009). Predicting recurrence of nonfunctioning pituitary adenomas. J Clin Endocrinol Metab.

[6] Wierinckx A, Auger C, Devauchelle P, Reynaud A, Chevallier P, Jan M (2007). A diagnostic marker set for invasion, proliferation, and aggressiveness of prolactin pituitary tumors. Endocr Relat Cancer.

[7] Freda PU, Chung WK, Matsuoka N, Walsh JE, Kanibir MN, Kleinman G (2007). Analysis of GNAS mutations in 60 growth hormone secreting pituitary tumors: correlation with clinical and pathological characteristics and surgical outcome based on highly sensitive GH and IGF-I criteria for remission. Pituitary.

[8] Katznelson L, Laws ER, Melmed S, Molitch ME, Murad MH, Utz A (2014). Acromegaly: an endocrine society clinical practice guideline. J Clin Endocrinol Metab.

[9] Cazabat L, Libà R, Perlemoine K, René-Corail F, Burnichon N, Gimenez-Roqueplo AP (2007). Germline inactivating mutations of the aryl hydrocarbon receptor-interacting protein gene in a large cohort of sporadic acromegaly: mutations are found in a subset of young patients with macroadenomas. Eur J Endocrinol.

[10] Clementi E, Malgaretti N, Meldolesi J, Taramelli R (1990). A new constitutively activating mutation of the Gs protein alpha subunit-gsp oncogene is found in human pituitary tumours. Oncogene.

[11] Livak KJ, Schmittgen TD (2001). Analysis of relative gene expression data using real-time quantitative PCR and the 2 (-Delta Delta C(T)) Method. Methods.

[12] Thapar K, Kovacs K, Scheithauer BW, Stefaneanu L, Horvath E, Pernicone PJ (1996). Proliferative activity and invasiveness among pituitary adenomas and carcinomas: an analysis using the MIB-1 antibody. Neurosurgery.

[B13] Landis CA, Masters SB, Spada A, Pace AM, Bourne HR, Vallar L (1989). GTPase inhibiting mutations activate the alpha chain of Gs and stimulate adenylyl cyclase in human pituitary tumours. Nature.

[14] Yang I, Park S, Ryu M, Woo J, Kim S, Kim J (1996). Characteristics of gsp-positive growth hormone-secreting pituitary tumors in Korean acromegalic patients. Eur J Endocrinol.

[B15] Mendoza V, Sosa E, Espinosa-de-Los-Monteros AL, Salcedo M, Guinto G, Cheng S (2005). GSPalpha mutations in Mexican patients with acromegaly: potential impact on long term prognosis. Growth Horm IGF Res.

[B16] Lania A, Spada A (2009). G-protein and signalling in pituitary tumours. Horm Res.

[17] Yasufuku-Takano J, Takano K, Morita K, Takakura K, Teramoto A, Fujita T (2006). Does the prevalence of gsp mutations in GH-secreting pituitary adenomas differ geographically or racially? Prevalence of gsp mutations in Japanese patients revisited. Clin Endocrinol (Oxf).

[18] Landis CA, Harsh G, Lyons J, Davis RL, McCormick F, Bourne HR (1990). Clinical characteristics of acromegalic patients whose pituitary tumors contain mutant Gs protein. J Clin Endocrinol Metab.

[B19] Shi Y, Tang D, Deng J, Su C (1998). Detection of gsp oncogene in growth hormone-secreting pituitary adenomas and the study of clinical characteristics of acromegalic patients with gsp-positive pituitary tumors. Chin Med J (Engl).

[20] Buchfelder M, Fahlbusch R, Merz T, Symowski H, Adams EF (1999). Clinical correlates in acromegalic patients with pituitary tumors expressing GSP oncogenes. Pituitary.

[21] Persani L, Borgato S, Lania A, Filopanti M, Mantovani G, Conti M (2001). Relevant cAMP-specific phosphodiesterase isoforms in human pituitary: effect of Gs(alpha) mutations. J Clin Endocrinol Metab.

[22] Chen M, Wang J, Dickerson KE, Kelleher J, Xie T, Gupta D (2009). Central nervous system imprinting of the G protein G(s)alpha and its role in metabolic regulation. Cell Metab.

[23] Picard C, Silvy M, Gerard C, Buffat C, Lavaque E, Figarella-Branger D (2007). Gs alpha overexpression and loss of Gs alpha imprinting in human somatotroph adenomas: association with tumor size and response to pharmacologic treatment. Int J Cancer.

[24] Hayward BE, Barlier A, Korbonits M, Grossman AB, Jacquet P, Enjalbert A (2001). Imprinting of the G(s)alpha gene GNAS1 in the pathogenesis of acromegaly. J Clin Invest.

[25] Fajardo-Montaãana C, Daly AF, Riesgo-Suárez P, Gómez-Vela J, Tichomirowa MA, Camara-Gómez R (2009). [AIP mutations in familial and sporadic pituitary adenomas: local experience and review of the literature] [article in Spanish]. Endocrinol Nutr.

[26] Tichomirowa MA, Barlier A, Daly AF, Jaffrain-Rea ML, Ronchi C, Yaneva M (2011). High prevalence of AIP gene mutations following focused screening in young patients with sporadic pituitary macroadenomas. Eur J Endocrinol.

[27] Chahal HS, Chapple JP, Frohman LA, Grossman AB, Korbonits M (2010). Clinical, genetic and molecular characterization of patients with familial isolated pituitary adenomas (FIPA). Trends Endocrinol Metab.

[28] Daly AF, Tichomirowa MA, Petrossians P, Heliövaara E, Jaffrain-Rea ML, Barlier A (2010). Clinical characteristics and therapeutic responses in patients with germ-line AIP mutations and pituitary adenomas: an international collaborative study. J Clin Endocrinol Metab.

[29] Cazabat L, Bouligand J, Salenave S, Bernier M, Gaillard S, Parker F (2012). Germline AIP mutations in apparently sporadic pituitary adenomas: prevalence in a prospective single-center cohort of 443 patients. J Clin Endocrinol Metab.

[30] Barlier A, Vanbellinghen JF, Daly AF, Silvy M, Jaffrain-Rea ML, Trouillas J (2007). Mutations in the aryl hydrocarbon receptor interacting protein gene are not highly prevalent among subjects with sporadic pituitary adenomas. J Clin Endocrinol Metab.

[31] Li Y, Zhou LP, Ma P, Sui CG, Meng FD, Tian X (2014). Relationship of PTTG expression with tumor invasiveness and microvessel density of pituitary adenomas: a meta-analysis. Genet Test Mol Biomarkers.

[32] Hunter JA, Skelly RH, Aylwin SJ, Geddes JF, Evanson J, Besser GM (2003). The relationship between pituitary tumour transforming gene (PTTG) expression and in vitro hormone and vascular endothelial growth factor (VEGF) secretion from human pituitary adenomas. Eur J Endocrinol.

[B33] Wierzbicka-Tutka I, Sokołowski G, Bałdys-Waligórska A, Adamek D, Radwańska E, Gołkowski F (2016). PTTG and Ki-67 expression in pituitary adenomas. Przegl Lek.

[34] Zhang X, Horwitz GA, Heaney AP, Nakashima M, Prezant TR, Bronstein MD (1999). Pituitary tumor transforming gene (PTTG) expression in pituitary adenomas. J Clin Endocrinol Metab.

[35] Trouillas J, Roy P, Sturm N, Dantony E, Cortet-Rudelli C, Viennet G (2013). A new prognostic clinicopathological classification of pituitary adenomas: a multicentric case-control study of 410 patients with 8 years post-operative follow-up. Acta Neuropathol.

[36] Thapar K, Scheithauer BW, Kovacs K, Pernicone PJ, Laws ER (1996). p53 expression in pituitary adenomas and carcinomas: correlation with invasiveness and tumor growth fractions. Neurosurgery.

[37] Salehi F, Agur A, Scheithauer BW, Kovacs K, Lloyd RV, Cusimano M (2009). Ki-67 in pituitary neoplasms: a review-part I. Neurosurgery.

[38] Hadzhiyanev A, Ivanova R, Nachev E, Elenkova A, Yaneva M, Zaharieva S (2014). Evaluation of prognostic utility of MIB-1 and p53 expression in pituitary adenomas: correlations with clinical behaviour and follow-up results. Biotechnol Biotechnol Equip.

[39] Sadeghipour A, Mahouzi L, Salem MM, Ebrahim-Nejad S, Asadi-Lari M, Radfar A (2017). Ki67 labeling correlated with invasion but not with recurrence. Appl Immunohistochem Mol Morphol.

